# A time-correlated single photon counting SPAD array camera with a bespoke data-processing algorithm for lightsheet fluorescence lifetime imaging (FLIM) and FLIM videos

**DOI:** 10.1038/s41598-024-56122-1

**Published:** 2024-03-27

**Authors:** Jakub Nedbal, Francesco Mattioli Della Rocca, Iveta T. Ivanova, Andrew Allan, Jeremy Graham, Richard Walker, Robert K. Henderson, Klaus Suhling

**Affiliations:** 1 Department of Physics, King’s College London, Strand, London, WC2R 2LS United Kingdom; 2 Institute for Integrated Micro and Nano Systems, The University of Edinburgh, Edinburgh, EH9 3FF United Kingdom; 3 Cairn Research, Graveney Road, Faversham, ME13 8UP United Kingdom; 4 Photon Force, Max Born Crescent, Edinburgh, EH9 3BF United Kingdom; 5 Present Address: Occuity, The Blade, Abbey Square, Reading, RG1 3BE United Kingdom; 6 Present Address: Europe Technology Development Centre, Sony Semiconductor Solutions - Sony Europe B.V., Trento, Italy

**Keywords:** Single photon avalanche diode (SPAD), Nonlinearity correction, Fluorescence lifetime imaging (FLIM), Selective-plane illumination microscopy, Algae, Giant unilamellar vesicle (GUV), FLIM video, Wide-field fluorescence microscopy, Light-sheet microscopy, Biological fluorescence

## Abstract

A wide-field microscope with epi-fluorescence and selective plane illumination was combined with a single-photon avalanche diode (SPAD) array camera to enable live-cell fluorescence lifetime imaging (FLIM) using time-correlated single-photon counting (TCSPC). The camera sensor comprised of $\text {192}\times \text {128}$ pixels, each integrating a single SPAD and a time-to-digital converter. Jointly, they produced a stream of single-photon images of photon arrival times with $\approx \text {38 ps}$ accuracy. The photon arrival times were subject to systematic delays and nonlinearities, which were corrected by a Monte-Carlo algorithm. The SPAD camera was then applied to FLIM where histogramming the resulting photon arrival times in each pixel resulted in decays compatible with common data processing pipelines for fluorescence lifetime analysis. The capabilities of the TCSPC camera-based FLIM microscope were demonstrated by imaging living unicellular photosynthetic algae and artificial lipid vesicles. Epi-fluorescence illumination enabled rapid fluorescence lifetime imaging of living cells and selective-plane illumination enabled 3-dimensional FLIM of stationary samples.

## Introduction

Fluorescence microscopy, with a history of over a hundred years^1^, can produce images of organisms or cells with high contrast and molecular specificity. Typical fluorescence microscopes generate image contrast based on the spatial distribution of light intensity collected from the fluorescent specimen. Fluorescence lifetime imaging (FLIM) microscopes produce images with fluorescence lifetime contrast, which deliver two main advantages: (1) Within a reasonable range, the fluorescence lifetime is independent of the often unknown fluorophore concentration. (2) FLIM can sense specimen properties not discernible in fluorescence intensity contrast. The fluorescence lifetime measured from a fluorophore in the specimen can be altered by the environment properties in its molecular vicinity. These environment properties may include the refractive index, pH, viscosity, polarity or the presence of nearby molecules^2–5^. The molecular environment affects fluorescence lifetime $\tau$ of the fluorophore because of the radiative $k_r$ and non-radiative $k_{nr}$ decay from its first singlet excited state.

1 $$\begin{aligned} \tau = \frac{1}{k_r + k_{nr}} \end{aligned}$$

The radiative decay rate $k_r$ is an intrinsic property of the fluorophore (via its extinction coefficient and emission spectrum) and the refractive index of its surroundings^6^. The non-radiative decay rate $k_{nr}$ is the sum of the rate constants for non-radiate decay processes like vibrational relaxation, intersystem crossing and quenching, and is typically a property of the interaction of the fluorophore’s excited state with its molecular surroundings. Each fluorophore responds differently to the environment changes and therefore it is possible to choose appropriate fluorophores to sense specific environment properties by FLIM.

The fluorescence lifetime can be measured in two principally different ways, in the frequency and time domains^7^. Frequency domain^8–11^ and time-gating time domain^12–17^ FLIM are most often done with wide-field detectors. However, time-correlated single photon counting (TCSPC) has the advantage over the aforementioned techniques in being the most photon efficient method for FLIM^18–23^. It also performs a direct and accurate measurement of the fluorescence decay shape. TCSPC-based FLIM is typically done with single-point detectors, which require raster scanning of the excitation laser beam, to produce microscope images^24,25^. Recently, wide-field (non-scanned) TCSPC-based FLIM microscopes and their applications have emerged thanks to new spatially- and time-resolved single-photon detectors^26–37^. Wide-field TCSPC FLIM has a number of advantages over raster-scanning FLIM. The image acquisition speed is not limited by the speed of the raster scanning mirrors. The microscope is optomechanically less complex and has lower fluorescence light losses, similar to any other camera-based microscopes. The new wide-field TCSPC detectors enable FLIM techniques like lightsheet microscopy, total internal reflection fluorescence, low-phototoxicity live-cell imaging or, in general, camera-based wide-field fluorescence microscopy^38^.

Lightsheet microscopy, or selective plane illumination microscopy, is widely used and was Nature Method’s method of the year 2014^39^. Its combination with FLIM allows functional imaging beyond the structural and morphological information that is obtained from fluorescence intensity-based lightsheet microscopy. There are some frequency-domain^10,40^ and time-gated^41,42^ lightsheet FLIM implementations, but very few TCSPC-based lightsheet FLIM implementations^29,43,44^. SPAD cameras are thus ideally placed to become detectors for TCSPC-based selective plane illumination FLIM^43,45^.

Fluorescence lifetime microscopy with single-photon avalanche diode (SPAD) arrays has been demonstrated in TCSPC mode^36,43,45–53^ but also with time-gated operation^14,17,37,54–56^, which has the advantage of a larger number of pixels and a higher fill factor. In the TCSPC regime, the photon arrival time is commonly measured by a time-to-digital converter (TDC). The TDC measures the time between the SPAD detecting the incident photon and the master reset signal provided by the pulsed laser source. The measurement is done by counting the number of ring oscillator increments, in this case, with the average step size of 38 ps^50,57^. The SPAD array image sensor used in this work had a dedicated TDC embedded in each of its $\text {192}\times \text {128}$ pixels. There are trade-offs between different SPAD architectures in terms of the fill-factor, data throughput, counting loss, manufacturing complexity, power consumption, etc. In the most advanced implementations, the SPAD array forms the top layer of a 3D stacked architecture, while the control and timing electronics reside on the bottom layer^58,59^. In the more common single-layer chips, SPADs and TDCs may occupy different areas to maximize the fill-factor, rather than having in-pixel TDCs. This could take the form of a silicon photomultiplier with embedded TDCs^60^ or grouping SPADs by columns, for instance, sharing the same TDC^61^ or a group of TDCs^62,63^. Other architectures are used for linear SPAD arrays, where several pixels can use one TDC to increase the light-sensitive area, and the TDCs are aligned along the length of the linear array^53,64^.

Here, each pixel has its own TDC for independent photon arrival timing and thus the parallel operation of 24576 TDCs enables a high overall photon count rate^52^. However, the chip design and manufacture introduced differences to the responses of the individual TDCs. These differences can be characterized as the differential nonlinearity (DNL) and the master reset timing delay. DNL is the local measure of TDC nonlinearity, it is the difference between the measured and ideal time increment between adjacent TDC bins. The master reset timing delay originates from the laser synchronization master reset pulse reaching the different TDCs at different moments due to the finite speed of the electronic signal traveling through the chip. These systematic errors introduced by the SPAD array image sensor pose a challenge to the downstream fluorescence lifetime analysis. They are not unique to SPAD array TDCs. Peak shifts at high conversion rates can occur in a time-to-analog converter (TAC) used for TCSPC, too^65^. However, existing TCSPC fluorescence lifetime analysis algorithms assume no delays between the decays in different pixels of the images and equally sized time bins in all pixels of the image. For accurate fluorescence lifetime analysis, using existing FLIM data analysis software, a new solution was therefore required.

Corrections of TDC artefacts were done in hardware and software in the past. Lookup tables were embedded into an field programmable gate array (FPGA) to correct TDC measurements in real time^66–71^. Software algorithms were developed to correct TDC nonlinearities. A neural network compensating for TDC nonlinearities was demonstrated to work with only a low number of calibration measurements^72^. In different pieces of work, the TDC response was linearized by using a periodically repeating eight bin photon-count scaling lookup table^47,48^. This was possible using the assumption that dominant nonlinearities resulted from the differences between the eight TDC ring oscillator elements. These algorithms offered an approximate solution to the TDC nonlinearity problem and still improved the accuracy of fluorescence lifetime determination. However, neither of these approaches dealt appropriately with the Poissonian distribution of photons and the fact that scaling the number of photons by a calibration value often resulted in the unrealistic non-integer number of photons.

This work describes a method to correct TDC nonlinearities and timing delay in a SPAD array with in-pixel TDCs which takes the Poissonian nature of the photon distribution into account. A Monte-Carlo synthesis of artificial photon arrival times based on the experimental data and calibration measurements was used to recreate corrected fluorescence decays for downstream FLIM analysis. The application of the method was demonstrated on a wide-field TCSPC fluorescence microscope equipped with the SPAD array camera. Image stills, videos of moving cells, and three-dimensional (3D) image volumes with fluorescence lifetime contrast were acquired with single-cellular plant organisms and artificial lipid vesicles.

## Results

### SPAD array TDC characterization

This work uses an existing SPAD array with $\text {192}\times \text {128}$ pixels, in-pixel TDCs, 13% optical fill factor, which was enhanced by cylindrical on-chip microlenses to 42%^50,57^. The SPAD array was produced in a 40 nm complementary metal-oxide semiconductor (CMOS) process to yield a $\approx \text {45\%}$ photon detection probability peaking at 500 nm^73^. Characterization measurements were performed on the SPAD array to provide information on the timing delay, which was the absolute time difference of the electronic laser synchronization pulse reaching each pixel, the absolute time width of each TDC bin and the dark count rate of each pixel. This was subsequently taken into account for the processing of the data. The timing delay was obtained from the measurement of the instrument response function with narrow light pulses ($\approx \text {100 ps}$) illuminating the sensor. The TDC bin width was obtained from a measurement of constant light illuminating the sensor which ensured uniformly distributed random photon arrival times. The dark count rate was measured with the sensor covered to exclude any light. With these calibration measurements, the Monte-Carlo algorithm could replace all photon arrival times acquired during an experiment with corrected synthesized photon arrival times. Subsequent binning of these virtual photon arrival times into histograms with equidistant bins recreated the fluorescence decays. The presented algorithm was not specific to a single sensor and application and can be applied to a wide range of time-resolved single-photon image and single-pixel sensors with TDCs or in microscopes and other optical systems. All measurements presented in this manuscript were acquired with a 1 ms exposure time.

#### Instrument response function is used to correct SPAD sensor timing delay

**Figure 1:**
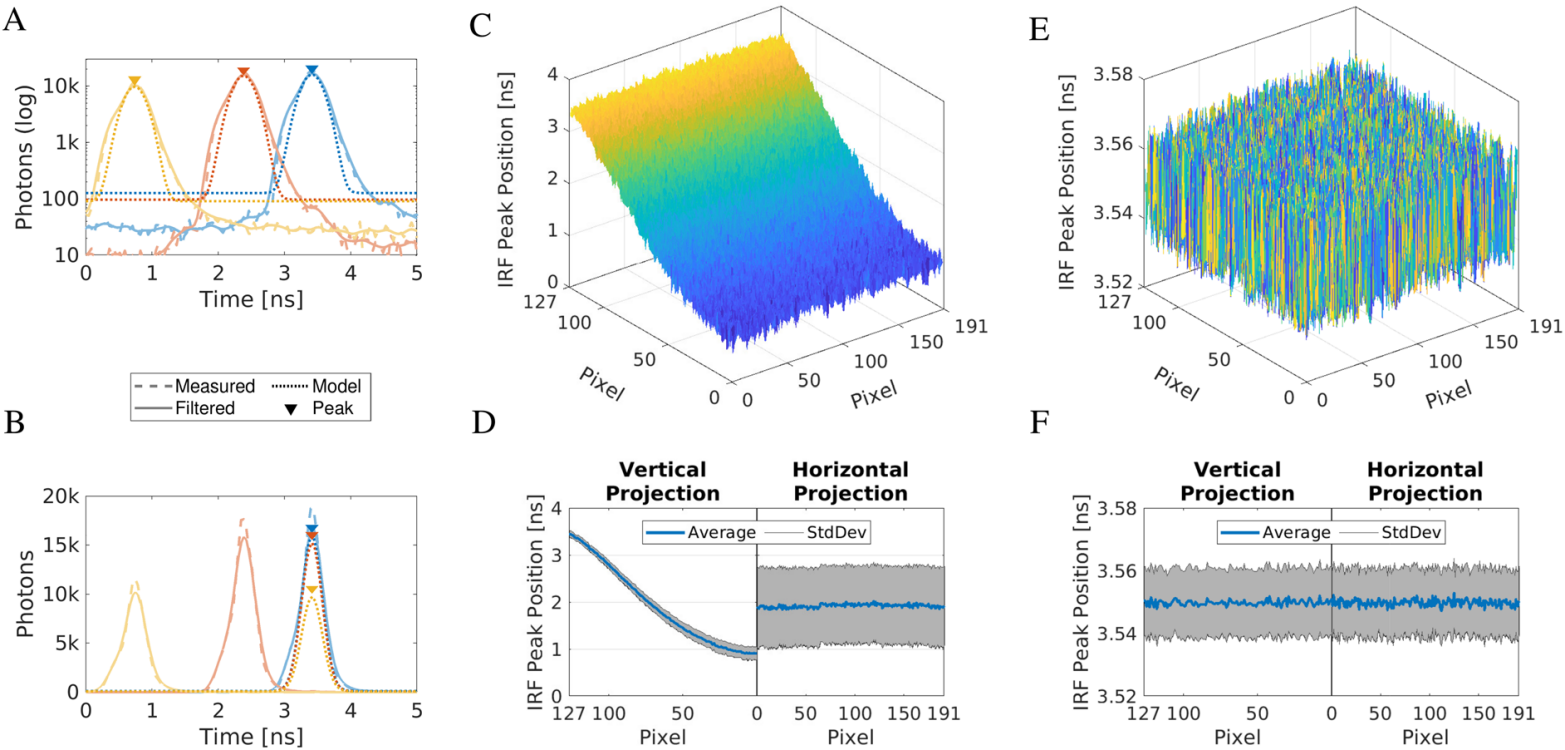
Instrument response function (IRF) characterization. (**A**) Example IRFs for three pixels in the SPAD array were plotted in dashed lines (blue: pixel [3, 140], red: [41, 140], yellow: [125, 140]) on a logarithmic vertical axis. They were spaced up to $\approx \text {3.5 ns}$ apart. The IRF shapes were filtered by a Gaussian filter with a kernel size of nine TDC bins (solid lines) and fitted with Gaussian models (dotted lines). The colored triangles highlight the peak positions of the IRF models. (**B**) The same as (**A**), but on a linear vertical axis, with the model IRFs translated to a common point in time as a result of the delay correction. (**C**) 2D map of measured IRF peak positions across the SPAD array. (**D**) Vertical and horizontal average projections (blue) of IRF peak positions were overlaid over their standard deviations range (gray). (**E**) 2D map of IRF peak positions after the correction. (**F**) Vertical and horizontal average projections (blue) and standard deviations (gray) of the corrected IRF peak positions.

The instrument response function (IRF) is the opto-electronic response of the SPAD camera sensor to pulsed light excitation. An IRF measurement was required to correct the timing delay of the SPAD sensor and for the calculation of the fluorescence lifetimes by exponential function fitting to the fluorescence decay curves. The most practical approach to measuring the IRF was by replacing the microscope sample with a fluorescent solution featuring a fluorescence lifetime much shorter than the width of the IRF. Since two different fluorophores with distinctive spectra (chlorophyll and di-4-ANEPPDHQ) and filter sets were used in the experiments, matching fluorescence samples for IRF measurements had to be found. An aqueous solution of Allura Red^74^ was used to measure the IRF for chlorophyll containing samples using the chlorophyll filter set. An IRF measured on a solution of fluorescein sodium salt quenched by I^-^ ions^75^ was used for di-4-ANEPPDHQ samples and the FITC filter set. The IRF peak position in time was calculated for each pixel by fitting its shape with a Gaussian function (Fig. 1A):

2 $$\begin{aligned} M(t) = A \exp \left( -\frac{(\mu - t)^2}{\sqrt{2} \sigma ^2}\right) + Z_0 \end{aligned}$$

*M*(*t*) is the shape of the fitted model function. $\mu$ and $\sigma$ are the mean and the standard deviation of the Gaussian, *A* is the amplitude scaling factor, and $Z_0$ is the background. The time positions of the peaks of the model *M*(*t*) defined the relative time delays between the TDCs in all pixels of the sensor (Fig. 1C, D). Shifting the peaks, so they overlapped (Fig. 1B), corrected the inherent time delay introduced in the sensor to produce the same timing response in each pixel (Fig. 1E, F). The effect of the correction was particularly clear in the vertical projection of the peak positions where the variation decreased from $\approx \text {3.5 ns}$ down to $\approx \text {0.02 ns}$. The shift correction process is described in detail in Sect. “Artefact correction algorithm” below. Our previously published work used an exponentially-modified Gaussian function as the model for the IRF^76^. Here, it was replaced with a Gaussian function, which improved the algorithm execution time and improved the peak position determination accuracy. It may seem counter-intuitive that the Gaussian model had higher accuracy of peak position determination compared to the exponentially-modified Gaussian model, which matched the IRF shape better. The reason was the relative simplicity of the Gaussian model and the reliable convergence to an accurate solution during in the iterative fitting algorithm.

#### Response to continuous light is used to correct SPAD sensor TDC nonlinearity

**Figure 2:**
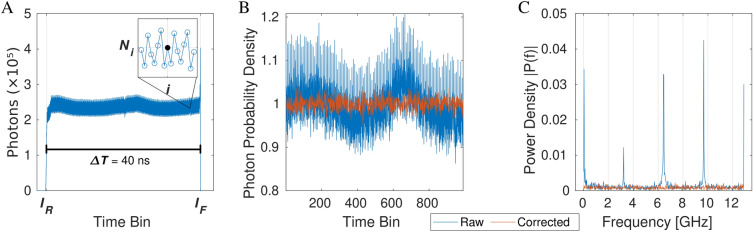
SPAD camera response to continuous light. (**A**) The solid blue line shows the number of photons counted in TDC time bins of a representative pixel under constant light illumination. The indices of the bins in the centers of the rising and falling edges are $I_R$ and $I_F$, respectively. The time between $I_R$ and $I_F$ is $\Delta T$, which is the period of the master clock reset oscillator. The inset shows a detail of the number of photons per bin $N_i$ around bin *i*. (**B**) Normalized DNL of the same pixel is shown in blue as measured by the TDC. In orange, is the same DNL after the TDC linearization. (**C**) A Fourier transform of the data in (**B**) shows distinctive peaks in the raw data (blue) and flat spectrum in the corrected data (orange).

Differential nonlinearity (DNL) is the difference between the measured and ideal time increments between adjacent TDC bins. The DNL was characterized by the response of the SPAD array sensor to constant (not pulsed) light illumination, which ensured photons were arriving randomly and uniformly distributed across the 50 ns active range of the TDCs. This range was set by the clock period of the 20 MHz oscillator attached to the synchronization input of the SPAD camera. The nonlinearities of the TDCs caused the deformation of this uniform distribution. An example of the photon distribution from a single pixel illuminated by a constant light and reset by a signal with a period of $\Delta T=\text {50 ns}$ is in Fig. 2A. The indices of the bins at the centers of the rising and falling edges are $I_R$, $I_F$, respectively. *i* is the index of an active TDC bin, which could vary between $I_R$ and $I_F$. $N_i$ is the number of photons counted in bin *i*. The width $W_i$ of each active TDC time bin *i* is proportional to the number of photons $N_i$ in the bin and the laser repetition period $\Delta T$ relative to the total number of photons.

3 $$\begin{aligned} W_i = \Delta T \frac{N_i}{\sum _{i=I_R}^{I_F} N_i} \end{aligned}$$

The accuracy of the TDC bin determination was dependent on the number of photons measured in each bin, as long as the clock source period $\Delta T$ was reliably known. The square root of the number of photons $N_i$ measured in a TDC bin *i* was also its standard deviation. The error of the bin width magnitude was consequently $\frac{1}{\sqrt{N_{i}}}$. This meant, to achieve 1% error in the determination of the bin width, required accumulating 10000 photons in a single TDC bin. The graph in Fig. 2A shows single bin photon count of $\approx \text {230}\,\text{000}$, equivalent to a relative error of $\approx \text {0.2\%}$. To achieve such high photon count in a single TDC bin, the measurements took several days to complete.

In summary, the IRF measured with a pulsed light source determined the relative time shifts between the different pixels. The photon density distribution, measured with constant light illumination, provided the absolute measure of time widths of the active TDC bins across the SPAD array.

### Artefact correction algorithm

**Figure 3:**
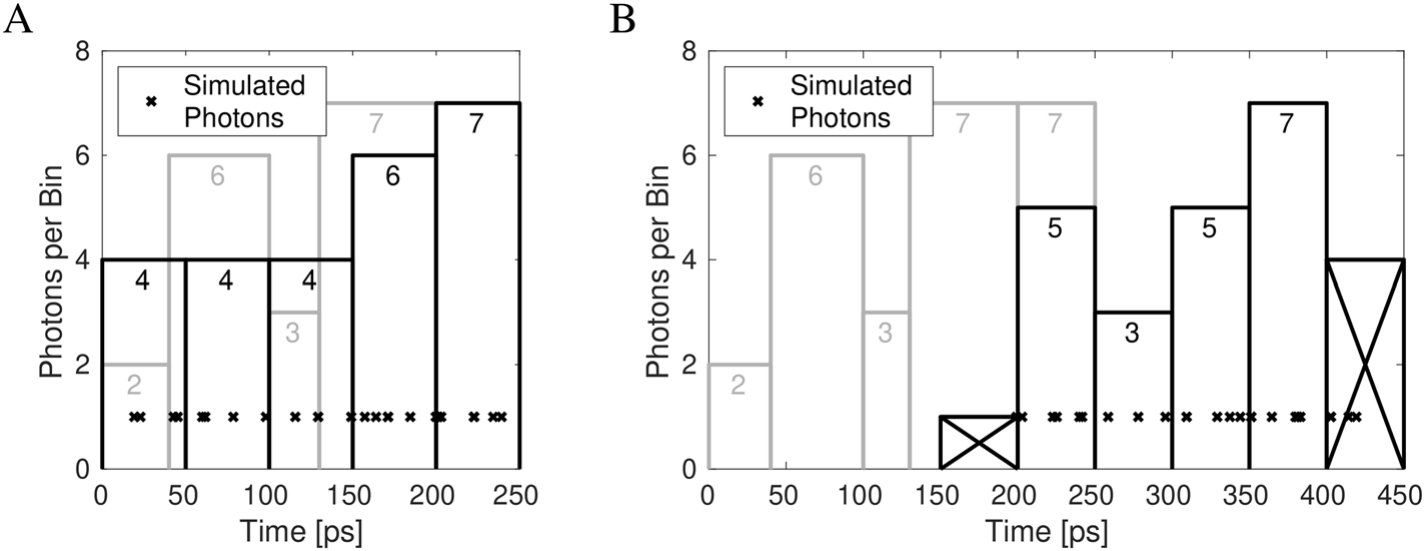
Explanation of TDC linearization through simulation. A simulation demonstrating TDC linearization principle used 25 randomly distributed photons and five TDC bins with different widths (40 ps, 60 ps, 30 ps, 70 ps, 50 ps). The widths of the light gray bars represent these histogram bin extents. Their heights represent the number of photons counted (2, 6, 3, 7, 7) in the respective bins (also written near the top of the bars). (**A**) Across the time span of each bin, uniformly randomly distributed photon arrival times were simulated for the number of photons in that bin. The simulated times are shown by the black crosses ($\times$). New equally-sized histogram bins were produced (50 ps width) and the numbers of simulated photons falling within each bin was counted (4, 4, 4, 6, 7). The equalized histogram bins were plotted as black bars, with the widths of 50 ps and their heights equivalent to the number of simulated photons falling in each. (**B**) The same simulation as (**A**), with the exception that the master reset timing delay was applied to all simulated photon arrival times. As a result, the arrival times of the simulated photons were shifted to counteract the SPAD-introduced timing delays. The same histogramming was performed with the bin width of 50 ps. The first and last bins were not used (crossed out), as they only partly covered the photon arrival time range due to the timing shift not being an integer multiple of the bin width. As a result, four bins containing 5, 3, 5, and 6 photons between 200 ps and 400 ps registered photons. Reproduced from^76^ with permission.

The Monte-Carlo linearization algorithm described in this section corrected the TDC master reset timing delays and differential nonlinearities inherent to the SPAD camera sensor. A link to its source code can be found in Table 1. The algorithm principle is explained with the help of a simulated experiment illustrated in Fig. 3. Five TDC bins and 25 photons are part of this simulation. The bins had different widths (40 ps, 60 ps, 30 ps, 70 ps, 50 ps) just like the SPAD camera TDC bins have different widths causing the differential nonlinearity. The widths of the five bins are equivalent to the widths of the grey bars in the illustration. Each bin recorded a certain number of photons in this simulation (2, 6, 3, 7, 7), which are reflected in the heights of the grey bars and the grey numbers near their tops. The exact photon arrival time within the bins cannot cannot be measured as the bin width is the smallest unit of time that can assigned to a photon recorded by the SPAD camera TDC or the simulation. The artefact correction algorithm overcomes this limitation by assigning randomly generated photon arrival times for each recorded photon uniformly distributed throughout the width of each bin. The time positions of these photons are marked by the black crosses in the illustration in Fig. 3A. These simulated photon arrival times are not the same as the actual photon arrival times impinging on the SPAD camera. Instead, in an approximation, they follow the same distribution as the real photons. Since the simulated photon arrival times are the results of random number generation, their time distribution would differ in each executions of the algorithm, similarly to the behavior of actual photons.

**Table 1:** Information on source code and data accompanying the manuscript.

Item	File name	File size	File type
Source Code	SPADlinearization.tgz	470 MB	.tgz (TGZ archive)
SPAD linearization code for processing the SPAD camera calibration and measurement data			
Source Code	rebinningSimulation.tgz	1.3 MB	.tgz (TGZ archive)
Fig. 4: Explanation of TDC linearization through simulation			
Source Code	graphs.tgz	720 MB	.tgz (TGZ archive)
Figs. 2, 3 & 5: Demonstration of linearization algorithm function and performance			
Source Code	algae_stills.tgz	116 MB	.tgz (TGZ archive)
Fig. 6: Data and source code producing microalgae FLIM image stills			
Source Code	Cvulgaris_timeseries.tgz	501 MB	.tgz (TGZ archive)
Fig. 7: Data and source code producing time series video of diffusing *C. vulgaris* cells			
Source Code	algae_lightsheet.tgz	1.2 GB	.tgz (TGZ archive)
Fig. 8: Data and source code producing videos of images stacks of algae cells			
Source Code	GUVs_lightsheet.tgz	498 MB	.tgz (TGZ archive)
Fig. 9: Data and source code producing videos of images stacks of GUVs			

To correct for the TDC nonlinearity, new ‘virtual’ TDC bins are designated with an identical 50 ps bin width. These new bins are marked by the black bars in Fig. 3A. All that remains is to count the number of photons, marked by the black crosses, in each of these new ‘virtual’ TDC bins. The heights of the black bars and the numbers near their tops (4, 4, 4, 6, 7) give the number of photons in each of the linearized ‘virtual’ bins.

To correct the TDC timing offset, a pixel-specific time delay, calculated from the IRF analysis (see Sect. “Instrument response function is used to correct SPAD sensor timing delay”), was added to the simulated photon arrival times. This is depicted in Fig. 3B by the black crosses being shifted to the right compared to Fig. 3A. The same process of new ‘virtual’ TDC bin designation is used to count the number of photons (5, 3, 5, 7) arriving in each of the identically-sized ‘virtual’ TDC bins. The first and last bins are ignored, as they only partially cover the range of possible photon arrival times.

The algorithm, when used on real experimental data, uses the numbers of photons $N_i$ recorded in the bins *i* with the known widths $W_i$ to create a ‘virtual‘ arrival time for each recorded photon during the experiment. The arrival times are shifted by a pixel-specific delay before being counted in pixel-specific histograms of equidistantly-spaced ‘virtual‘ TDC bins. The resulting histograms are equivalent to the measured time distribution of the fluorescence intensity without the nonlinearity and time delay artefacts introduced by the sensor.

#### The performance of the artefact correction algorithm

The linearity correction performance and repeatability were verified in an experiment in which the data for characterization and for testing were acquired months apart at similar room temperatures. Figure 1C, E show the timing offset maps calculated from the IRF before and after the correction. Figure 1D, F display their average projections and standard deviations along the vertical and horizontal directions. The images demonstrate the clear and consistent correction of the timing offsets across the entire array.

Figure 2B displays the photon probability density for a single pixel, calculated from the calibration measurement with a constant light illumination. The photon probability density is the number of photons in each TDC bin normalized by division with the average number of photons in all TDC bins of the pixel. The raw data are shown by the blue line and the corrected data by the orange line. The photon probability density plot of the raw data showed much higher variability compared to the corrected data. The raw data photon probability density featured oscillations, as evident from the distinctive peaks its Fourier transforms in Fig. 2C. The oscillations were consistent with the eight-step ring oscillator in the pixel TDC^50,57^. In contrast, the Fourier transform of the photon probability density function of the corrected data was uniformly distributed. This means that the oscillations in the SPAD sensor DNL were entirely suppressed by the correction algorithm and the remaining fluctuations were entirely down to the stochastic photon counting noise.

**Figure 4:**
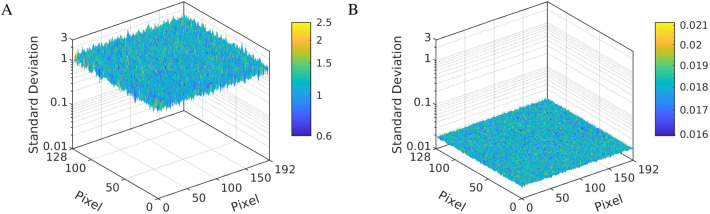
Performance of TDC linearization across the whole SPAD array. (**A**) Standard deviations of the photon density distributions in each pixel of the array were normalized by division with the square root of the total number of photons counted in given pixel and plotted as a 3D surface map. The average value of the normalized standard deviation was $\approx \text {1.2}$. The pixels near the lower and upper edges of the sensor showed a considerably lower value. (**B**) The standard deviations were normalized in the same way for the linearized data. It resulted in a similar value for all pixels of the array, which was $\approx \text {64}$ times lower compared to the nonlinearized data (average standard deviation of $\approx \text {0.018}$).

This result proved that that nonlinearities were successfully corrected in the single arbitrarily-chosen TDC from the array of $\text {192}\times \text {128}$ pixels. To prove this was the case for the all pixels of the sensor, the standard deviations of the photon density distribution were normalized by division with the square root of the total photons in each pixel. 2D maps of the results before and after the linearization were compared in Fig. 4. The normalized standard deviations varied between 0.12 and 2.5 across the array for the raw data from the SPAD array (Fig. 4A). The linearization decreased the normalized standard deviation to a comparable value for all pixels of the array and ranging between 0.015 and 0.022 (Fig. 4B). This demonstrates that the correction process was reliable across the entire sensor.

The execution time of the linearization depended on the number of photons in the measurement and the computer specification. For illustration, the linearization of an experimental dataset comprising of $\approx 1.5\times 10^9$ photon measurements took $\approx 12\,\text {s}$ to complete on a laptop with a quad-core i7-4810MQ processor and 32 GB random access memory (RAM). This execution time did not include the extra time required to load the calibration and experimental data, to fit the decays, and to save the the output files. The execution time would increase significantly if there was insufficient RAM available.

### Experimental verification of the SPAD array use in microscopy

The use of the sensor in microscopy was demonstrated on imaging experiments with microalgae and artificial lipid vesicles. The autofluorescence lifetime from chlorophyll was measured in two species of microalgae, *C. vulgaris* and *D. quadricauda* in three different imaging modes. Image stills of the cells with fluorescence lifetime contrast were produced using the SPAD camera alongside transmission images acquired on a standard CMOS camera. Time series videos of freely diffusing cells with intensity-weighted fluorescence lifetime contrast were acquired to demonstrate the feasibility of 1 s per frame rapid FLIM with low illumination intensity. Finally, 3D images of agarose-embedded cells were acquired to demonstrate the feasibility of selective-plane illumination microscopy. All experiments involved a comparison of fluorescence lifetime in unperturbed cells and cells treated with the photosynthesis inhibitor DCMU. To demonstrate the SPAD camera for FLIM in the most commonly used spectral range (green emission), giant unilamellar vesicles were labeled with a lipid order-sensitive dye di-4-ANEPPDHQ and imaged in the selective plane illumination mode.

#### Image stills of autofluorescence lifetime in algae

The chlorophyll A fluorescence lifetime is highly variable and depends on its interaction with other molecules (Eq. 1) in the cells, notably the reaction center of photosystem II^77^. Photosystem II, which converts the absorbed photon energy into electrochemical potential, will shorten the chlorophyll A fluorescence lifetime. Conversely, when photosystem II is unable to utilize the absorbed photon energy energy, chlorophyll A increases its lifetime. Fluorescence lifetime is therefore an important marker of the ability of the organism to process the absorbed light energy.

**Figure 5:**
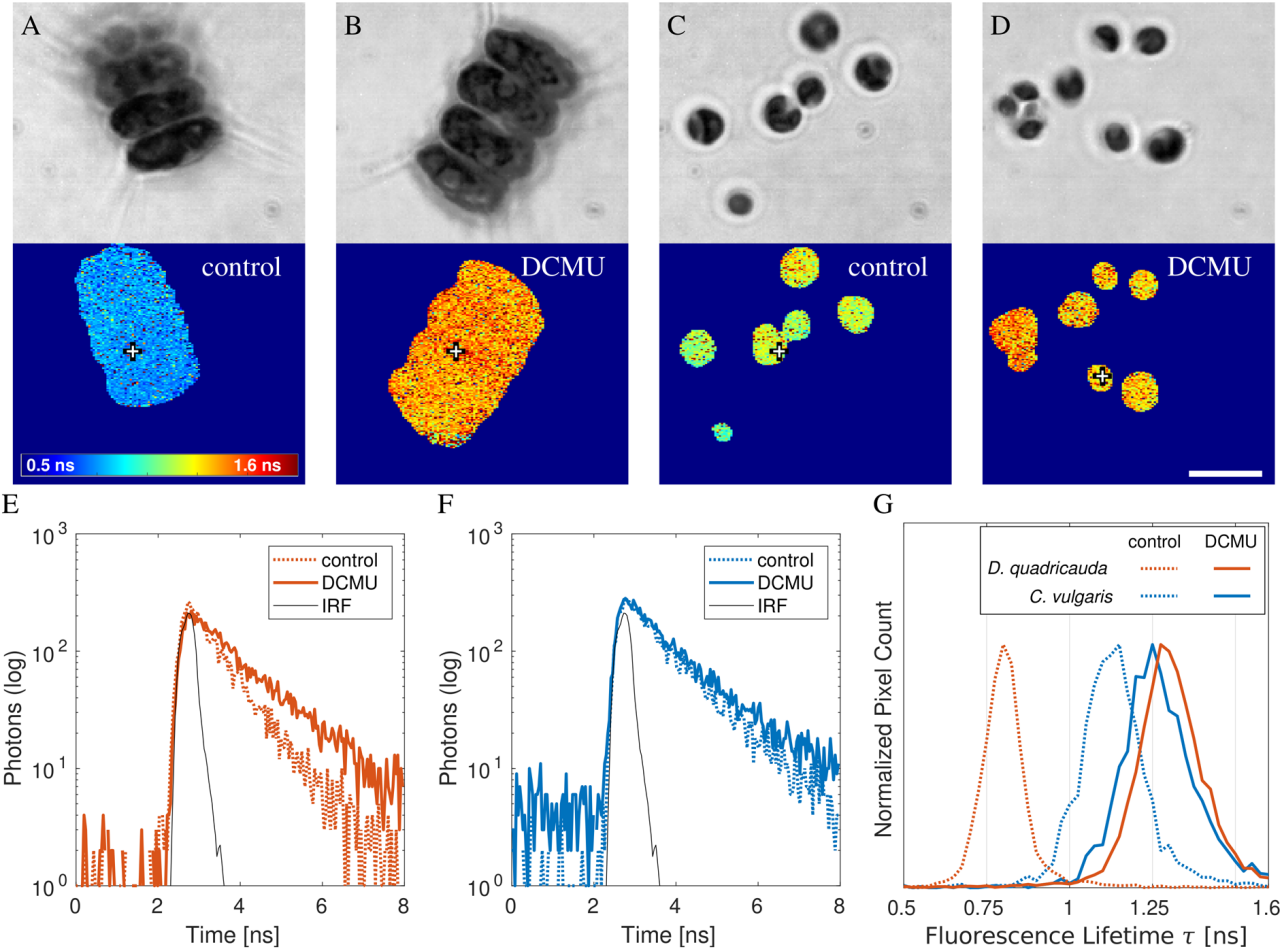
Autofluorescence lifetime imaging stills of algae. (**A**–**B**) Transmission images of the microalgae are in the top row. Their fluorescence lifetime contrast images are in the bottom row. The fluorescence lifetime contrast range (0.5–1.6 ns) is the same in all four panels with the palette colorbar displayed in (**A**). The crosses show the positions of representative pixels, for which the decays are in (**E**, **F**). *D. quadricauda* are in (**A**, **B**, **E**). *C. vulgaris* are in (**C**, **D**, **F**). Samples (**A**, **C**) are the control cells that were only treated with DMSO and exhibit a shorter lifetime. Samples (**B**, **D**) were incubated with the photosynthesis inhibitor DCMU in DMSO and display a longer lifetime. (**E**, **F**) The representative fluorescence decays of control cells (dotted lines) and DCMU-treated cells (solid lines) are displayed with the measured IRF (black line, scaled to range). (**G**) Normalized histograms show the fluorescence lifetime distributions in the non-zero pixels of the images. Scale bar $\text {10}\,\upmu \text {m}$. Reproduced from^76^ with permission.

Chlorophyll A autofluorescence lifetime images of *C. vulgaris* and *D. quadricauda* were acquired on the microscope using the SPAD camera and a band-pass emission filter centered at 667 nm. The cells in these experiments were treated with either DCMU in the solvent DMSO, or DMSO only, as a control. The DCMU blocked the electron transport chain in photosystem II by competing for plastoquinone binding site Q_B_^78^. The chlorophyll A fluorescence lifetime was expected to increase in the presence of DCMU due to the blocking of the photosystem II electron transport chain. This was indeed observed in the fluorescence lifetime images of the cells (Fig. 5) treated with and without DCMU. This difference was more pronounced in *D. quadricauda* (Fig. 5A, B, E) with a fluorescence lifetime histogram shift from around 0.8 ns (untreated) to around 1.3 ns (treated with DCMU) compared to *C. vulgaris* (Fig. 5C, D, F), whose fluorescence lifetime histogram shifted only from around 1.2 ns (untreated) to around 1.25 ns (treated with DCMU, as shown in Fig. 5G). The results of this experiment are presented in three ways: Fig. 5A–D images of fluorescence lifetime contrast, Fig. 5E, F representative fluorescence decays, Fig. 5G and normalized histograms of fluorescence lifetime distributions in the cells. Overall, the FLIM data demonstrated that DCMU increased the fluorescence lifetime of chlorophyll A as expected.

#### Rapid frame rate videos of autofluorescence lifetime in algae

**Figure 6:**
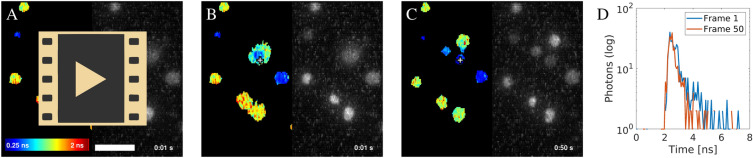
Time series FLIM of freely diffusing algae cells. (**A**) Video of fluorescence lifetime acquired under epi-fluorescence illumination at one frame per second and showing diffusing *C. vulgaris* cells. The cells were a mixture of half treated with DCMU and half untreated. The treated cells had a longer lifetime (appear more red). (**B**) First frame of the video shows the fluorescence lifetime contrast on the left and the fluorescence intensity contrast on the right. The cross in the middle highlights the pixel [96, 48]. The underlying fluorescence decay in this pixel is in (**D**, blue line). (**C**) The same as (**B**), but showing the last frame of the video. The cross is in the same position, but over a different cell, which diffused into the location during the video acquisition. The underlying fluorescence decay is in (**D**, orange line). (**A**–**C**) Images were produced with $\text {3}\times \text {3}$ spatial binning during the fluorescence lifetime analysis. (**D**) The decays show a representative pixel sampled in the first frame (blue) and the last frame (orange). The decays were made by $\text {3}\times \text {3}$ spatial binning (in X and Y) and binning of 2 in T (time). Scale bar $\text {10}\,\upmu \text {m}$. Fluorescence lifetime range 0.25 ns –2.0 ns. Reproduced from^76^ with permission.

Rapid and accurate FLIM measurement was possible with the SPAD array camera, thanks to its pixels operating in parallel. This was demonstrated on a combined suspension of *C. vulgaris* cells untreated and pre-treated with DCMU. The cells were moving by Brownian motion in the field of view of the microscope. The example video is in Fig. 6A. It was made of 50 frames with the rate of one frame per second. The stills of the first and last frames are in Fig. 6B, C. Representative decays from the first and the last frame in the same pixel, but covering different cells, are shown in Fig. 6D. The data shows that it was possible to capture *C. vulgaris* cells in motion while measuring their fluorescence lifetime distribution to distinguish cells with different photosynthetic activities.

#### 3D imaging videos of autofluorescence lifetime in algae

**Figure 7:**
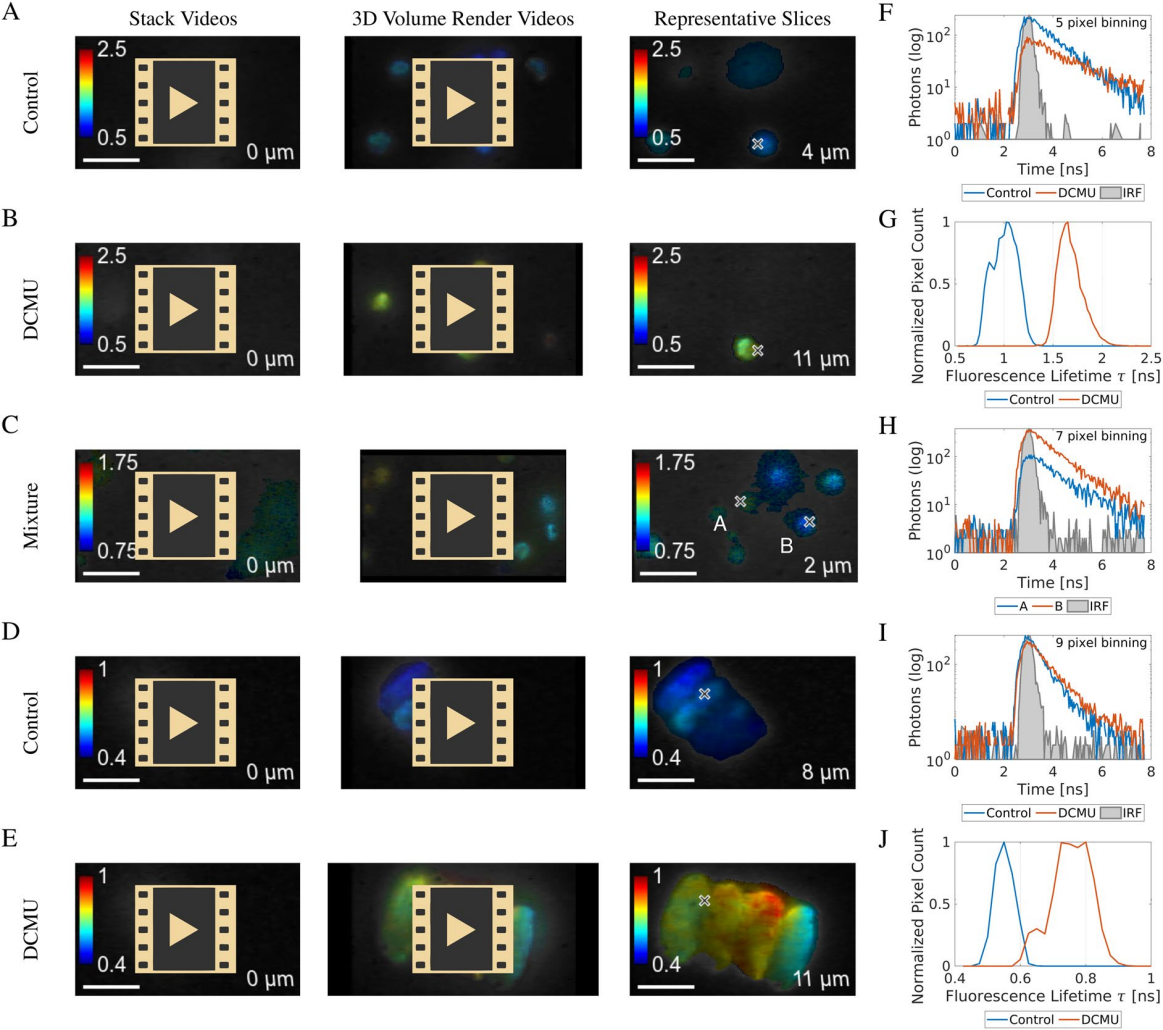
Selective plane illumination fluorescence lifetime volume images of algae cells. (**A**–**E**) Panels are made of three columns labeled at the top. The first column contains URL links to image stack fly-through videos of immobilized cell suspension. The second column contains links to 3D volume render videos of the same image stacks. The third column contains example images from the stacks with crosses highlighting the points from which the representative decays are shown in (**F**, **H**, **I**) alongside the measured IRF in gray. (**A**) Videos and image of *C. vulgaris* cells that have not been treated with DCMU and thus show a lower intrinsic fluorescence lifetime. (**B**) Videos and an image of *C. vulgaris* cells treated with photosynthesis inhibitor DCMU. (**C**) Videos and an image of a mixture of *C. vulgaris* cells pre-treated with DCMU and untreated control cells. The fluorescence lifetime contrast in the mixture video (**C**) was lower than in the pure samples (**A**, **B**). (**D**) Videos and image of *D. quadricauda* cell coenobium not treated with DCMU and thus showing a lower intrinsic fluorescence lifetime. (**E**) Videos and image of *D. quadricauda* cell coenobium treated with DCMU. (**F**) Representative fluorescence decays taken from the same coordinate (marked by a cross) in the images in (**A**, **B**). (**G**) Histograms of fluorescence lifetime distribution in the 3D stacks of images in (**A**, **B**). (**H**) Representative fluorescence decays taken from two different coordinates, labelled (**A**,** B**) in (**C**). (**I**) Fluorescence decays taken from the same coordinate (marked by a cross) in the images in (**D**, **E**). (**J**) Histograms of fluorescence lifetime distribution in the 3D stacks of images in (**D**, **E**). Scale bars $\text {10}\,\upmu \text {m}$. Numbers in the lower-right corners of the videos and images mark the relative axial position of each image stack. (**A**−**C**) no binning, (**D**, **E**) binning $7\times 7$ pixels.

3D live cell FLIM is rarely performed due to the need to meet opposing experimental requirements of low excitation levels and rapid acquisition. The parallel processing of the SPAD array, when combined with selective plane illumination, enabled 3D imaging at a high frame rate and low light dose, commensurate with live cell imaging. To exemplify this, algae cells treated with DCMU and/or untreated control cells were embedded in agarose and subjected to 3D FLIM microscopy using the Mizar Tilt illumination system with a lightsheet thickness of $4.3\,\upmu \text {m}$^79^. The acquisition speed was 20 seconds per image with the images axially spaced by 1 $\upmu$m. *C. vulgaris* cells were treated with DCMU, left untreated, or in a mixture of treated and untreated cells. *D. quadricauda* cells were treated with DCMU or untreated. The cell suspensions were embedded in agarose (Fig. 9) and imaged with $\text {1}\,\upmu \text {m}$ axial spacing. The resulting image stack data was rendered in an intensity weighted fluorescence lifetime contrast. The videos showed axial image stack fly-throughs and 3D projections rotated around the vertical axis of the volume in Fig. 7. The presented data showed the feasibility of 3D selective plane illumination imaging of cells with fluorescence lifetime contrast using the SPAD array camera. (Fig. 7A, D) Control cells, not treated with DCMU, showed consistently lower and similar fluorescence lifetime ($\approx \text {1.0 ns}$ for *C. vulgaris* and $\approx \text {500 ps}$ for *D. quadricauda*) compared to (Fig. 7B, E) the DCMU-treated cells ($\approx \text {1.7 ns}$ and $\approx \text {800 ps}$ for *D. quadricauda*). Videos of a mixture of *C. vulgaris* cells containing DCMU-treated and untreated cells (Fig. 7C) showed cells with different characteristic fluorescence lifetimes, although the fluorescence lifetime contrast was less pronounced than between the pure populations. This was due to the lack of DCMU in the embedding medium unlike in the pure DCMU-treated cells, which had a working concentration of DCMU present during the image acquisition. Consequently, the DCMU was loosing its efficacy over time, decreasing the fluorescence lifetime. The experiments were characterized by graphs in addition to the videos. (Fig. 7F−J) Representative decays from the experiments in areas highlighted by crosses in Fig. 7A, B are in Fig. 7F, in two areas of Fig. 7C, highlighting a cell exposed to DCMU and a cell not treated with DCMU, are in Fig. 7H and from Fig. 7D, E are in Fig. 7I. Histograms of fluorescence lifetimes in pixels from the videos in Fig. 7A, B are in Fig. 7G and from the videos in Fig. 7D, E are in Fig. 7J. The histograms show a good separation between the different cells according to their exposure to DCMU. Overall, the live cell experimental results on the 3D imaging of microalgae cells were consistent with the image still experiments and the time-series experiments in (Figs. 5 and 6).

#### 3D imaging videos of giant unilamellar vesicles fluorescence lifetime

**Figure 8:**
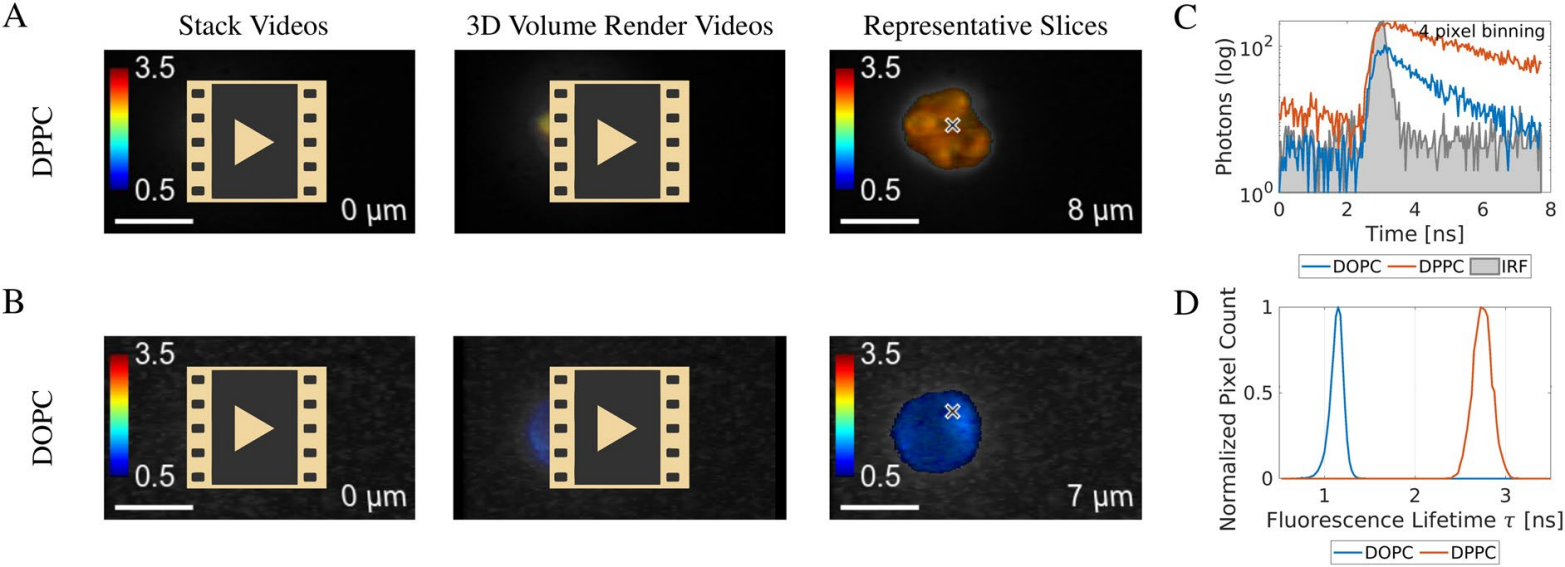
Selective plane illumination fluorescence lifetime volume images of GUVs. (**A**, **B**) Panels are made of three columns titled at the top. The first column contains URL links to image stack fly-through videos of immobilized GUVs. The second column contains links to 3D volume render videos of the same image stacks. The third column contains representative images from the stacks with crosses highlighting the points from which the representative decays were produced (**C**). (**A**) Videos and an image of a GUV cluster prepared from a DPPC/cholesterol mixture. The di-4-ANEPPDHQ in this membrane exhibits a longer fluorescence lifetime ($\approx \text {2.7 ns}$). (**B**) Videos and an image of a GUV prepared from DOPC. The di-4-ANEPPDHQ in this membrane exhibits a much shorter fluorescence lifetime ($\approx \text {1.1 ns}$). (**C**) Representative fluorescence decays taken from the same coordinate (marked by a cross) in the images in (**A**, **B**). The decays contain 5670 counts (A) and 1417 counts (B), respectively. (**D**) Histograms of fluorescence lifetime distribution in the 3D stacks of images in (**A**, **B**). Scale bars $\text {10}\,\upmu \text {m}$. Numbers in the lower-right corners of the videos and images mark the relative axial position of each image stack.

To demonstrate 3D FLIM in the green fluorescence emission spectral region, giant unilamellar vesicles (GUVs) were prepared from two different lipid mixtures. They were labelled with di-4-ANEPPDHQ fluorescent dye, embedded in agarose and imaged in the selective plane illumination mode with the SPAD camera and a band-pass emission filter centered at 535 nm. The fluorescent dye was sensitive to the packing state of the lipid bilayer and thus the composition of the membrane. This feature was exploited by creating the GUVs from different constituents with the expectation of observing different fluorescence lifetimes. The GUV membranes were created from DOPC, giving a disordered lipid phase, or a 3:1 mixture of DPPC and cholesterol, giving an ordered phase. Videos, images, fluorescence decays and fluorescence lifetime histograms are presented in Fig. 8A–D. The DOPC-based GUV in Fig. 8B shows a much shorter fluorescence lifetime ($\approx \text {1.1 ns}$, see Fig. 8C) compared to the DPPC/cholesterol GUVs in Fig. 8A ($\approx \text {2.7 ns}$) as illustrated in the fluorescence lifetime histogram in Fig. 8D. There were 5670 counts and 1417 counts in the fluorescence decays in Fig. 8A, B, respectively. For 20000 1 ms exposures, the count rate was therefore 71 Hz and 284 Hz in these pixels, respectively. With a laser excitation repetition rate of 78  MHz, this is well below a count rate where photon pile-up becomes a concern^31,80^.

The videos and images of the GUVs did not show sharp contours due to their small size compared to the lightsheet thickness of $4.3\,\upmu \text {m}$^79^ and the spherical aberration caused by the use of the available oil immersion objective with the aqueous sample. Yet, the SPAD camera combined with the selective plane illumination allowed 3D imaging of the GUVs with fluorescence lifetime contrast using a commonly used FITC filter set.

## Discussion

The main goal of this work was to develop and demonstrate a procedure for correcting systematic errors in TCSPC imaging devices and apply it to SPAD-based FLIM microscopy. Although the method was applied to a specific SPAD camera^50,57^, it should be applicable to other TCSPC devices and applications, too. Calibration measurements were performed to characterize the TDC bin width and timing delays. A Monte-Carlo algorithm used this calibration data to resample the measured photon arrival times into linearized and corrected photon distribution histograms ready for downstream analysis.

The results demonstrated the reliable function of the algorithm with the time-resolved SPAD camera under the same imaging and laboratory conditions. Calibration and test measurements were taken months apart with no noticeable decline in the calibration accuracy. The corrected TDC linearity and timing delays remained consistent across all pixels of the array.

The SPAD array camera and the presented data analysis pipeline enabled wide-field FLIM to achieve the high accuracy and photon efficiency of TCSPC without raster scanning^38^. The image acquisition was much faster compared to conventional TCSPC scanning microscopes. These microscopes typically operate with laser powers in the range of $\upmu \text {W}$^31^ focused to a diffraction limited spot $(<\text {1}\,\upmu \text {m}^\text {2})$. In contrast, the peak sample irradiance in the wide-field FLIM presented here was 3–4 orders of magnitude lower at $\text {160 W}\text {m}^{-\text {2}}$—less than the peak solar irradiance at the surface of Earth ($\approx \text {1000 W}\text {m}^{-\text {2}}$)^81^. Scaled to the entire field of view of the camera ($\text {1160 }\upmu \text {m}^2$), only 180 nW of laser power irradiated the visible part of the sample. This advantage was especially beneficial in live cell imaging, where phototoxicity is an important consideration^82,83^. However, it was even more important in the cellular chlorophyll A fluorescence imaging described in this report, as exposure to excitation light drives unwanted changes in the photosynthetic samples^77^. Despite such low irradiance, the camera was fast in practical imaging, too. One second per frame acquisition time was sufficient to produce high quality fluorescence lifetime contrast videos with a single-exponential model and spatial binning of only $\text {3}\times \text {3}$ pixels. This remarkable speed for TCSPC FLIM was possible thanks to the parallel TDC operation. The data acquisition speed could be increased further by using samples less sensitive to excitation light, increasing the excitation laser power or increasing the frame rate. The SPAD camera proved versatile and simple to use in microscopy. It could be connected to an output port on any epi-fluorescence microscope equipped with a pulsed laser light source and used with the described analysis pipeline. Thus, TCSPC wide-field FLIM could potentially become more affordable and accessible, compared to the common raster scanning approaches.

The SPAD array image sensor featured a high effective fill factor (42%) due to its optimized pixel design and the microlenses manufactured onto its surface^50,57^. The resulting stripe artefacts and the excessive dark count rate in some pixels negatively impacted on the fluorescence intensity image quality. Appropriate image scaling, pixel sensitivity normalization, image segmentation utilizing only every second image column, and spatial interpolation were implemented to overcome these image imperfections. Microscopic images with fluorescence intensity contrast of satisfactory quality could thus be produced while benefiting from the high image sensor fill factor.

The opportunities for improvement to the calibration procedure and the correction algorithm are discussed below. The described linearization process assumed photons were arriving uniformly randomly distributed across the individual TDC bins. This was a valid assumption for illumination with constant light. It remained reasonable even for fluorescence decays, as each bin was only $\approx \text {38 ps}$ wide. However, especially for fast decaying fluorescence or instrument response function measurement, this assumption is not strictly accurate. To adapt to the decaying signal, a local derivative of the number of photons per bin or the fitted fluorescence decay model could be used to perform the photon resampling with a decaying probability density distribution. In contrast, photon pile-up^31,80^ had a negligible effect because the photon count rate per pixel was maintained below 1 kHz, considerably lower than the laser pulse repetition frequency of 78 MHz. Although using a lower repetition rate would be desirable and technically feasible for the GUV experiments (Fig. 8C), but the fluorescence lifetime contrast between the two samples was sufficient (Fig. 8D) to demonstrate the capability of the technique.

It is known that clock sources can cause nonlinearities in TCSPC^84^. The clock source used for the TDC histogram bin width measurement with constant light (buffered crystal oscillator at 20 MHz) and the clock source used for the IRF and the experimental data measurements (pulsed laser at 78 MHz) were different and residual clock-induced nonlinearities may have remained in the linearized data. It would be advantageous to either verify that the nonlinearities were the same for both clock sources or acquire all data with the same clock source. The verification experiment showed that the correction of the nonlinearities did not deteriorate between measurements taken months apart (Fig. 1C, E). However, there is a possibility of temperature-dependent effects on the TDC nonlinearity. The sensor temperature will increase with higher irradiance due to the higher activity of the SPAD and TDC circuits. It is possible that limits of the nonlinearity correction could be found if the sensor irradiance differs much between the calibration measurement and the experiment.

Cross-talk between TDCs due to power-supply fluctuation caused by TDC activity are unlikely to have a measurable effect on the nonlinearities. This is due to the low photon count rate ($<1\,\text {kHz per pixel}$) compared to the laser repetition rate of 78 MHz. Even with the full array being illuminated, most laser pulse periods will not have more than one TDC active at a time, meaning that no cross-talk between active TDCs can happen. Should occasionally more than one TDC operate simultaneously, the effect on the bin size change is estimated to be $\approx 0.01\,\%$. This estimate is based on the current draw of a single TDC being $\approx 100\,\upmu \text {A}$, the power supply resistance being $\approx 1\,\Omega$ and its voltage 1.1 V.

The peak sample irradiance was very low compared to conventional TCSPC, but still relatively high for microalgae or other plant cells ($\text {600}\, \upmu \text {mol photons}\,\,\, \text {m}^{-2}\,\,\text {s}^{-1}$). It could have lead to measurement-induced fluorescence lifetime increase due to photosystem II closure and possibly other effects^77^. Nevertheless, the addition of the photosynthesis inhibitor DCMU, which disrupts the photosynthetic electron transport chain, clearly led to a chlorophyll fluorescence lifetime increase, as shown in Fig 7. The effect of the measurement light on the photosynthetic cells could be investigated in the future to balance a sufficiently high data acquisition speed, acceptable signal-to-noise, and undetectable changes to the fluorescence lifetime in the intact cells.

This work solved the problem of transforming the SPAD array output data with systematic errors into error-free data for downstream analysis. This added an additional step into the experimental pipeline. An alternative solution would be to use the calibration measurements and adjust the fluorescence lifetime analysis algorithm to work with the nonlinear and time-shifted input data. This would simplify the workflow and improve the data processing speed. This work is serving as a bridge before such algorithms are developed or the correction is performed in the hardware^66–71^.

The SPAD camera and data analysis pipeline were demonstrated on FLIM. However, the detector and the software are agnostic in relation to the application and downstream analysis tools. They could be directly applied to other potential TCSPC applications requiring linear timing response, for example time-resolved fluorescence anisotropy^85,86^ or other wide-field FLIM modalities such as total internal reflection FLIM^30^.

## Methods

### SPAD array camera

The image acquisition was done on a QuantICAM SPAD array camera with $\text {192}\times \text {128}$ pixels (University of Edinburgh, UK). Each pixel contains a dedicated SPAD and a TDC. The image sensor incorporated microlenses, extending its effective fill factor to 42%^50,57^. The SPAD camera featured a USB 3.0 connection for data transfer and communication with a computer. The camera TDC master reset input signal was connected to the pulsed monitor output of the laser through an RG-316 coaxial cable (Farnell, Leeds, UK). The camera was mounted in a custom housing made from alumide by 3D printing (i.materialise, Leuven, Belgium). The 3D computer-aided design (CAD) model of the camera case, designed in Onshape, is available for viewing and editing^87^. The camera case was fixed to the optical table by a magnetic kinematic base (KB-75M, Thorlabs, Ely, UK) for easy alignment-free mounting and removal. The camera was operated by software written in MATLAB (Mathworks, Cambridge, UK)^88^. The principle of operation of the camera is explained in the following paragraph.

During its operation, the camera kept receiving an electronic master reset pulse synchronization clock signal from the pulsed excitation laser. Whenever a photon was absorbed and triggered an avalanche breakthrough in the photodiode of a particular pixel, it started a time counter in the pixel TDC. This counter was stopped by the subsequent master reset pulse clock rising edge. The photon detection event disabled the pixel for the remainder of the exposure time, which was set to 1 ms for all experiments. After the end of each exposure, the TDC values were read from all pixels and transferred to the computer to give a single frame of photon arrival times. Each frame consisted of pixels which contained the last registered TDC value or a zero value in pixels that had not detected a photon during the exposure time. Fluorescence decay measurements were obtained from repeated exposures combined into histograms of photon arrival times in all pixels. The histograms were automatically produced at the end of the measurement from 20000 repeated 1 ms exposures in case of the selective plane illumination experiments. In the case of the time series experiments, histograms were only produced during the post processing, allowing arbitrary choice of video frame rate. The presented videos had 1 second frame rate with each frame produced from 1000 1 ms exposures.

### Microscope

A Nikon TE2000 U microscope body was used with a CFI Apo TIRF 60XC oil objective (both Nikon UK, Surbiton, UK) and a chlorophyll filter cube (ET445/30x excitation filter, T470lpxr dichroic beamsplitter, ET667/30m emission filter, Cairn Research, Faversham, UK) or a standard a standard fluorescein isothiocyanate (FITC) filter cube (Nikon UK). The microscope was enclosed in a light-tight box made from aluminium struts, connection elements (KJN, Leicester, UK), and 6 mm PVC sheets (RS Components, Corby, UK). Excitation light was provided by a supercontinuum laser SuperK Extreme (FIU-15, NKT, Birkerød, Denmark). The infrared component of the laser output spectrum was removed by reflection off two extended hot mirrors (46-386, Edmund Optics, York, UK)^89^ and passed through an absorptive KG3 glass (FGS900-A, Thorlabs, Ely, UK). The light was filtered further by a 498 nm short-pass filter (FF01-498/SP-25, Laser2000, Huntingdon, UK) and coupled into a single mode fiber optic patch cable (P3-460B-FC-2, Thorlabs). The output light from the fiber was collimated by a 100 mm achromatic lens and focused into the microscope objective back focal plane by a 250 mm achromatic lens (both Thorlabs). The QuantICAM SPAD camera sensor was positioned in the image plane outside the right output port of the microscope. This port received 80% of the light collected by the objective with the remaining 20% sent to the eyepiece. A CMOS camera (DCC-1545M, Thorlabs) was attached to the microscope eyepiece through a $\text {1}\times$ C-mount eyepiece adapter to generate transmission images. Transillumination light was generated using a custom light source made of 24 LEDs (OIS-170-675-X-T, RS Components) with an emission peak of 675 nm arranged in a ring concentric with the objective.

Selective plane illumination microscopy was performed on the microscope described above, but additionally expanded with the Mizar Tilt illuminator (Cairn Research). The excitation light was supplied to the Mizar Tilt illuminator using the same single-mode fiber described above. The axial microscope stage movement and the SPAD camera image acquisition were synchronized by a custom MATLAB script to acquire the image stacks.

The experiments used the laser operating at a 78 MHz pulse repetition rate. The photosynthetically active radiation (PAR) at the sample was $\text {600}\, \upmu \text {mol photons}\,\,\, \text {m}^{-2}\,\,\text {s}^{-1}$ at a wavelength centered around 445 nm during the time series experiments. The PAR value could not be measured or reliably estimated for the selective plane illumination experiments due to the complex illumination pattern of the lightsheet microscope created by the interference of four wavefronts and the insufficient space for inserting the available power meter photodiode.

The cells were adapted to the excitation light for about 10 minutes prior to the measurement. The time series measurements took $\approx \text {50 s}$ (50000 single-photon frames with 1 ms exposure time each). The selective plane illumination measurements took several minutes to complete, depending on the height of the image stack, with 20000 single-photon frames (1 ms) acquired for each image.

### Calibration measurements

TDC nonlinearity was measured by illuminating the SPAD array camera sensor with a constant light source provided by a green LED (LGT67K-H2K1-24-Z, Farnell, Leeds, UK) from a distance of 15 mm. The LED was supplied from a 2 mA constant current source. The TDC master reset signal was provided by a buffered 20 MHz temperature-compensated crystal oscillator. Around 200000 photons were acquired for each TDC bin in every pixel.

The master reset timing delay measurement, equivalent to an instrument response function (IRF) measurement, was conducted on the microscope with the SPAD array camera attached. Pulsed light excitation was provided by the supercontinuum laser. The samples were fluorescent solutions with very short intrinsic fluorescence lifetime made of quenched fluorescein^75^ or Allura Red^74^ and used with FITC or chlorophyll fluorescent filter sets, respectively. Freshly prepared solutions of saturated fluorescein sodium salt (46960, Sigma-Aldrich, Gillingham, UK) and saturated sodium iodide (217638, Sigma-Aldrich) in aqueous 100 mM sodium diphosphate buffer (10783445, Fisher Scientific, Loughborough, UK) were used as the sample for use with the FITC filter set. This solution had $\ll \text {100 ps}$ fluorescence lifetime^75^, meaning the measured signal was dominantly formed by the instrumental response. Fresh 0.5 mM Allura Red (38213-25mg, Sigma-Aldrich) solution in deionized water was prepared to measure the instrument response function with the chlorophyll filter set. This solution had an even shorter intrinsic fluorescence lifetime of $\approx \text {11 ps}$^74^.

### Data processing pipeline

The described algorithm was implemented in Mathworks^®^ MATLAB using the *DIPimage* library^90^. The source code is available for download^88^, see Table 1. This code imported the calibration and experimental data produced by the SPAD camera and produced image cytometry standard (ICS) files for downstream fluorescence lifetime analysis in TRI2^91,92^. Bayesian single-exponential fluorescence lifetime estimation was used to obtain the presented results. TRI2 produced ICS files with fluorescence lifetime, amplitude, and intensity images that were further processed in MATLAB to correct the imaging artefacts of the SPAD camera. Imperfect alignment of microlenses on the SPAD array image sensor surface caused patterning in the photon count (fluorescence intensity) images. To overcome this line pattern across the fluorescence intensity images, the values in the pixels of the dim columns were replaced by interpolation using the nearest-neighbor method. Furthermore, the intensity values for the 20% of pixels with the highest dark count rate were replaced with interpolated values. This led to some loss of detail in the images, but overall resulted in clearer images devoid of artefacts stemming from the varying performance of the different pixels of the SPAD array. Based on these processed fluorescence intensity images, image areas containing cells or GUVs were segmented by the application of automatic threshold and edge smoothing using area erosion and growth filters. The resulting images with fluorescence lifetime contrast were also processed before display. Only data from $\text {80}\,\%$ of the pixels with the lowest dark count rate $(<\text {31 Hz, median 8.3 Hz})$ were used to produce the resulting images. The fluorescence lifetime values in the remaining 20% (hot) pixel were obtained by nearest-neighbor interpolation. Images were scaled using a cubic interpolation by a factor of $2\times$ in the horizontal direction to correct for the rectangular pixel pitch in the SPAD array.

### Microscope specimens

All samples were prepared for microscope imaging in glass bottom dishes (80807, Thistle Scientific, Glasgow, UK). Unicellular algae *C. vulgaris* and *D. quadricauda* (both CCALA, Třeboň, Czech Republic) were cultured as described previously^93^. Briefly, cells were grown at room temperature in 1/2SŠ inorganic medium supplemented with 10 mM $\textrm{NaHCO}_{3}$ on a bottom-illuminated orbital shaker operating at 100 revolutions per minute. The cells were grown in daily cycles of photosynthetically active radiation of $\text {200}\, \upmu \text {mol photons}\,\,\, \text {m}^{-2}\,\,\text {s}^{-1}$ for 14 h followed by 10 h of darkness. Cells were imaged in glass bottom dishes (80807, Thistle Scientific, Glasgow, UK) in 1/2SŠ medium. The density of cultures per area of coverslip were $\text {5}\times \text {10}^\text {5}\,\text {cm}^{-\text {2}}$ for *C. vulgaris* and $\text {1}\times \text {10}^\text {5}\,\text {cm}^{-\text {2}}$ for *D. quadricauda*. The samples were supplemented with either 0.1% (v/v) dimethyl sulfoxide (DMSO) (276855, Sigma-Aldrich) as a control or 10 $\upmu$M working dilution of 3-(3,4-dichlorophenyl)-1,1-dimethylurea (DCMU) (D2425, Sigma-Aldrich) in DMSO, which disrupts the electron transport chain and inhibits photosynthesis. For fast video data acquisition, samples were prepared by mixing *C. vulgaris* cells treated and non-treated with DCMU. Equal volumes of the cell suspensions were incubated for 30 min with DCMU or DMSO only. They were washed twice in the 1/2SŠ medium involving centrifugation at 3000 relative centrifugal force (RCF) for 2 min to remove the DCMU. Both samples were resuspended in 1/2SŠ medium, combined, and imaged immediately.

For selective plane illumination microscopy *C. vulgaris* cells were embedded in 0.5% low-melting temperature agarose to restrict their movement. 1% (w/w) agarose (A9414, Sigma-Aldrich) solution was prepared in 1/2SŠ medium by dissolving agarose powder, or melting a stock solution, at 70 ^∘^C and cooling it down to 42 ^∘^C in separate water baths before use. The agarose was mixed in 1:1 (v/v) ratio with either water, to make clear agarose well wall, or the cell suspension to embed the cells.

**Figure 9:**
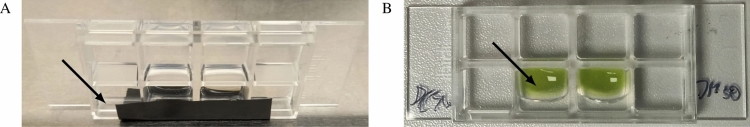
Embedding algae cells in agarose. (**A**) An outer well wall made of transparent agarose was prepared before embedding cells in agarose for the illumination beam to pass with minimal scattering. The well was prepared by setting $\text {300}\,{\upmu }\text {l}$ of low-melting temperature agarose in a temporary well at the side of a glass-bottom well dish created by a piece of tape (arrow), sealing part of the well opening. (**B**) A suspension of algae in agarose (arrow) was added to the well and set before imaging. The clear agarose well wall is visible below the green algae.

Before embedding any cells, an outer agarose wall was produced inside the glass bottom dish. The selective plane illumination beam passed through this clear agarose wall with minimal scattering before impinging on the cell suspension. The agarose wall preparation method is illustrated in Fig. 9. A tape was used to seal an outer part of the well opening. The well was placed sideways with the tape at the bottom. The resulting temporary well created by the tape was filled with $\text {300}\,{\upmu }\text {l}$ of freshly mixed 1:1 (v/v) ratio of solubilized agarose at 42 ^∘^C and room-temperature deionized water (Fig. 9A). The agarose was left to set before adding cell suspension in agarose. Cells were washed by centrifugation at 3000 RCF for 2 minutes and resuspended in fresh medium either containing DCMU (in DMSO at 10 $\upmu$M working dilution) or an equivalent amount of DMSO without DCMU as a control. These suspensions were mixed with equal volume of molten agarose at 42 ^∘^C and transferred into the prepared wells (Fig. 9B). The agarose in the imaging wells was briefly ($<\text {5 min}$) cooled down in refrigerator before immediate imaging on the selective plane illumination microscope. A third sample was prepared from a mixture of DCMU-treated and untreated cells. After a 10-minute incubation, cells were washed, resuspended in fresh medium, immobilized in agarose, as described above, and imaged.

Giant unilamellar vesicles (GUVs) were synthesised using the gentle hydration method^94^. Two types of GUVs, representing different packing states of the lipid bilayer, were created: A disordered phase bilayer was made from homogeneous 1,2- dioleoyl-sn-glycero-3-phosphocholine (DOPC) (850375P, Sigma-Aldrich) and an ordered phase bilayer from a mixture of 1,2- dipalmitoyl-sn-glycero-3-phosphocholine (DPPC) (850355P, Sigma-Aldrich) and cholesterol (CH200, Molecular Dimensions, Rotherham, UK). A 10 mM chloroform solution of DOPC and a mixture of 7 mM DPPC and 3 mM cholesterol was prepared in 2 ml glass vials (10236703, Fisher Scientific). The vials were cleaned with chloroform (366927, Sigma-Aldrich) and dried with steady flow of dry nitrogen gas. di-4-ANEPPDHQ (D36802, Fisher Scientific) was added to each vial to a final concentration of $\text {20 }\upmu \text {M}$. From these stock solutions, $\text {100 }\upmu \text {l}$ were transferred to a clean vial and left to evaporate overnight in a fume hood to leave a thin lipid film at the bottom. Meanwhile, oxygen-depleted deionized water was prepared by bubbling nitrogen gas for 20 min. The next day the lipid films were gently hydrated with 0.5 ml of deionized water. The water was added by a slow steady flow from the pipette tip along the inner wall of the vials. The aqueous suspensions were left overnight for spontaneous vesicle formation to take place. The DOPC sample was left at room temperature, while the DPPC/cholesterol sample was incubated in a 49 ^∘^C water bath. The next day, $\text {300}\,\upmu \text {l}$ of each was mixed with equal volume of molten 1% agarose at 42 ^∘^C and added to the glass bottom dishes to solidify before imaging.

## Data Availability

The data supporting this article is openly available from the King’s College London research data repository, KORDS, at 10.18742/c.6217211. The files containing the source code and data used to produce the figures and videos in this manuscript are in Table 1. The source code is distributed with a BSD 2.0 license and the data with a CC BY license.
